# Determinantes Comuns da Pressão Arterial e do Nível de Testosterona

**DOI:** 10.36660/abc.20240234

**Published:** 2024-08-07

**Authors:** Yusuf Ziya Şener, Alexandr Ceasovschih

**Affiliations:** 1 Hacettepe University Faculty of Medicine Internal Medicine Department Ankara Turquia Hacettepe University Faculty of Medicine – Internal Medicine Department, Ankara – Turquia; 2 University of Medicine and Pharmacy Faculty of Medicine Iasi Romênia Faculty of Medicine – "Grigore T. Popa" University of Medicine and Pharmacy, Iasi – Romênia

**Keywords:** Pressão Arterial, Hipertensão, Testosterona, Obesidade, Hipogonadismo, Autonegligência

**Caro Editor,**

Lemos o artigo publicado por Negretto et al.,^1^ com grande interesse. Os autores relataram que a deficiência de testosterona está presente em 26,3% dos pacientes com hipertensão arterial e o nível de testosterona diminui com o aumento da idade e do índice de massa corporal.^1^ O papel da testosterona na hipertensão e os fatores que afetam tanto a pressão arterial quanto o nível de testosterona são tópicos interessantes e negligenciados na prática diária.

O consumo de álcool é frequente em todo o mundo e tem efeitos tanto nos níveis de testosterona como na pressão arterial. Foi demonstrado que o consumo de altas doses de álcool produz uma diminuição da pressão arterial até 12 horas, mas aumenta a pressão arterial 12 horas após o consumo.^2^ Apesar de existirem dados conflitantes sobre o impacto do álcool no nível de testosterona, o consenso geral sustenta que o consumo crônico de álcool resulta em deficiência de testosterona.^3^ Portanto, seria melhor se o estado do consumo de álcool tivesse sido levado em consideração.

Os hormônios tireoidianos desempenham um papel principal no controle de diversas vias metabólicas e tanto o hipotireoidismo quanto o hipertireoidismo podem levar ao aumento da pressão arterial.^4^ O hormônio tireoidiano aumenta os níveis de globulina de ligação aos hormônios sexuais e de testosterona total. O hipotireoidismo causa resposta reduzida ao hormônio liberador de gonadotrofina (GnRH), resultando na diminuição dos níveis de testosterona livre.^5^ Assim, as funções da tireoide desempenham um papel significativo na interação entre a pressão arterial e os níveis de testosterona.

Os betabloqueadores são comumente usados em doenças cardiovasculares e foram atualizados recentemente para opções de tratamento de primeira linha na hipertensão.^6^ Vários estudos estabeleceram que os betabloqueadores diminuem o nível de testosterona e causam disfunção erétil.^7^ Portanto, pensamos que seria melhor se o uso de betabloqueadores tivesse sido avaliado no presente estudo.

Para concluir, a testosterona desempenha um papel significativo na pressão arterial e deve-se ter em mente que existem vários fatores que têm impacto na interação entre a pressão arterial e os níveis de testosterona.
