# Supplementary material for: Determinantes Comuns da Pressão Arterial e do Nível de Testosterona
Source: Arq Bras Cardiol. 2024 Aug 7;121(7):e20240234. [Article in Portuguese] doi: 10.36660/abc.20240234 (PMC11495812; doi:10.36660/abc.20240234)
Supplement: Translation

# Translation: Common Determinants of Blood Pressure and Testosterone Level

**DOI:** 10.36660/abc.20240234i

**Authors:** Yusuf Ziya Şener, Alexandr Ceasovschih

**Affiliations:** 1 Ankara Turkey Hacettepe University Faculty of Medicine – Internal Medicine Department, Ankara – Turkey; 2 Iasi Romania Faculty of Medicine – "Grigore T. Popa" University of Medicine and Pharmacy, Iasi – Romania

**Keywords:** Arterial Pressure, Hypertension, Testosterone, Obesity, Hypogonadism, Self-Neglect

**Dear Editor,**

We read the article published by Negretto et al.^1^ with great interest. The authors reported testosterone deficiency is present in 26.3% of the patients with arterial hypertension and testosterone levels decrease by increasing age and body mass index.^1^ The role of testosterone in hypertension and factors affecting both arterial blood pressure and testosterone level are interesting and overlooked topics in daily practice.

Alcohol consumption is frequent worldwide and it has effects on both testosterone levels and blood pressure. It was demonstrated that high-dose alcohol consumption yields a decrease in blood pressure up to 12 hours however it raises blood pressure 12 hours after consumption.^2^ Despite there are conflicting data about the impact of alcohol on testosterone levels, general consensus supports that chronic alcohol consumption results in testosterone deficiency.^3^ Therefore it would be better if the status of alcohol consumption had been taken into consideration.

Thyroid hormones play a main role in the control of several metabolic pathways and both hypothyroidism and hyperthyroidism may lead to increased blood pressure.^4^ Thyroid hormone increases sex hormone binding globulin and total testosterone levels. Hypothyroidism causes reduced response to gonadotropin-releasing hormone (GnRH) resulting in decreased free testosterone levels.^5^ Thus, thyroid functions play a significant role in the interaction between blood pressure and testosterone levels.

Beta-blockers are commonly used in cardiovascular diseases and upgraded to the first-line treatment option for hypertension recently.^6^ Several studies established that beta-blockers decrease the level of testosterone and cause erectile dysfunction.^7^ Therefore, we think that it would be better if the use of beta-blockers had been assessed in the present study.

To conclude, testosterone plays a significant role in blood pressure and it should be kept in mind that several factors have an impact on the interaction between blood pressure and testosterone levels.
