# Supplementary material for: Determinantes Comuns da Pressão Arterial e do Nível de Testosterona
Source: Arq Bras Cardiol. 2024 Aug 7;121(7):e20240234. [Article in Portuguese] doi: 10.36660/abc.20240234 (PMC11495812; doi:10.36660/abc.20240234)
Supplement: Related texts

# Reply: Carta-resposta

**Authors:** Leandra Analia Freitas Negretto, Nelson Rassi, Leonardo Ribeiro Soares, Amanda Bueno Carvalho Saraiva, Maria Emília Figueiredo Teixeira, Luciana da Ressurreição Santos, Ana Luiza Lima Souza, Paulo Cesar B. Veiga Jardim, Weimar Kunz Sebba Barroso de Souza, Thiago de Souza Veiga Jardim

**Caro Editor,**

Agradecemos a leitura diligente do artigo o qual foi o resultado de um estudo de vida real em que foram avaliados os valores de Testosterona Total (TT) em homens hipertensos, buscando a prevalência da deficiência do hormônio (TT < 300nd/dL) bem como a associação com diversos fatores considerados de proeminente relevância.^1^

Embora o consumo de álcool seja de fato uma ameaça à saúde cardiovascular.^2^ a sua pesquisa tornaria este estudo demasiadamente desafiador, haja vista que seria necessário o emprego de um instrumento de avaliação padronizado^3^ para mensurar e documentar a magnitude do uso do álcool.

Além disso, é de amplo conhecimento que indivíduos que fazem uso abusivo de álcool muitas vezes precisam estar acompanhados quando interrogados a esse respeito, o que teria o potencial de comprometer a avaliação da saúde sexual,^4^ por ser este um tema que pode ferir o pudor do paciente.

A despeito do fato de a função tireoidiana ter relevância nos diversos cenários de doença cardiovascular,^5^ ela não faz parte do hall de exames de rotina do indivíduo hipertenso.^6–8^ O estudo foi desenvolvido em um centro de atenção terciária de uma Universidade Federal de medicina do Brasil, realizado sem fonte financiadora, de modo que os exames coletados foram aqueles considerados cruciais para a propedêutica do indivíduo com hipertensão (HA).

Os betabloqueadores são de fato anti-hipertensivos de primeira linha no tratamento da HA mundo afora, porém quando comparado às demais classes de anti-hipertensivos amplamente usadas na HA não há diferença significativa sobre o potencial impacto negativo sobre a saúde sexual masculina.^9^

Já o emprego da Espironolactona, por sua vez, foi considerado uma variável pertinente neste estudo, por ser notadamente um fármaco com ação anti-androgênica,^10^ o que poderia enviesar o estudo, caso não fosse procurado ativamente.

A despeito do fato de os níveis de hormônio masculino serem especialmente relevantes em contextos de aumento do risco cardiovascular, eles ainda não compõe a propedêutica do indivíduo com HA, assim como a abordagem de seus possíveis causadores de deficiência.

Desta maneira agradecemos as considerações apontadas, as quais podem ser de grande utilidade em estudos futuros tão necessário para trazer luz a este tema ainda tão negligenciado.

## References

[1] Negretto LA, Rassi N, Soares LR, Saraiva AB, Teixeira ME, Santos LR (2024). Testosterone Deficiency in Hypertensive Men: Prevalence and Associated Factors. Arq Bras Cardiol.

[2] Day E, Budd JHF (2019). Alcohol use disorders and the heart. Adiction.

[3] Cardoso DP, Oliveira D, Antunes B, Saraiva R, Angus K, Galardo E (2022). Portuguese Validated Versions of the Alcohol Use Disorders Identification Test: A Systematic Review Protocol. Acta Med Port.

[4] Morley JE, Charlton E, Patrick P, Kaiser FE, Cadeau P, McCready D (2000). Validation of a screening questionnaire for androgen deficiency in aging males. Metabolism.

[5] Cappola AR, Desai AS, Medici M, Cooper LS, Egan D, Sopko G (2019). Thyroid and Cardiovascular Disease: Research Agenda for Enhancing Knowledge, Prevention, and Treatment. Circulation.

[6] Barroso WK, Rodrigues CI, Bortolotto LA, Gomes MA, Brandão AA, Feitosa AD (2021). Brazilian guidelines of hypertension - 2020. Arq Bras Cardiol.

[7] Arnett DK, Blumenthal RS, Albert MA, Buroker AB, Goldberger ZD, Hahn EJ (2019). 2019 ACC/AHA Guideline on the Primary Prevention of Cardiovascular Disease: A Report of the American College of Cardiology/American Heart Association Task Force on Clinical Practice Guidelines. Circulation.

[8] Whelton PK, Carey RM, Mancia G, Kreutz R, Bundy JD, Williams B (2022). Circulation Society of Hypertension Blood Pressure/ Hypertension Guidelines: Comparisons, Reflections, and Recommendations. Circulation.

[9] Farmakis IT, Pyrgidis N, Doundoulakis I, Mykoniatis I, Akrivos E, Giannakoulas G (2022). Effects of Major Antihypertensive Drug Classes on Erectile Function: a Network Meta-analysis. Cardiovasc Drugs Ther.

[10] Bagnoli V, Fonseca A, Cezarino P, Fassolas G, Arie J, Baracat E (2010). Hormonal treatment of acne based on evidence. Femina.

# Reply

**Authors:** Leandra Analia Freitas Negretto, Nelson Rassi, Leonardo Ribeiro Soares, Amanda Bueno Carvalho Saraiva, Maria Emília Figueiredo Teixeira, Luciana da Ressurreição Santos, Ana Luiza Lima Souza, Paulo Cesar B. Veiga Jardim, Weimar Kunz Sebba Barroso de Souza, Thiago de Souza Veiga Jardim

**Dear Editor,**

We would like to thank you for diligently reading the article, which was the result of a real-life study in which Total Testosterone (TT) values were evaluated in hypertensive men, seeking the prevalence of hormone deficiency (TT < 300nd/dL) as well as the association with several factors considered to be of prominent relevance.^1^

Although alcohol consumption is indeed a threat to cardiovascular health,^2^ your research would make this study too challenging, given that it would be necessary to use a standardized assessment instrument^3^ to measure and document the magnitude of alcohol use.

Furthermore, it is widely known that individuals who abuse alcohol often need to be accompanied when questioned in this regard, which could potentially compromise the assessment of sexual health,^4^ as this is a topic that can hurt the patient's modesty.

Even though thyroid function is relevant in different scenarios of cardiovascular disease,^5^ it is not part of the routine exams for hypertensive individuals.^6–8^ The study was developed in a tertiary care center at a Federal University of Medicine in Brazil, carried out without a funding source so that the tests collected were those considered crucial for the workup of individuals with hypertension (AH).

Beta-blockers are in fact first-line antihypertensives in the treatment of AH around the world, however, when compared to other classes of antihypertensives widely used in AH, there is no significant difference regarding the potential negative impact on male sexual health.^9^

The use of Spironolactone, in turn, was considered a pertinent variable in this study, as it is notably a drug with anti-androgenic action,^10^ which could bias the study if it were not actively sought.

Although male hormone levels are especially relevant in contexts of increased cardiovascular risk, they still do not form part of the workup for individuals with AH, as well as the approach to possible causes of deficiency.

In this way, we would like to thank you for the considerations mentioned, which can be of great use in future studies, which are so necessary to shed light on this topic that is still so neglected.
